# Leveraging multiple approaches for the detection of pathogenic deep intronic variants in developmental and epileptic encephalopathies: A case report

**DOI:** 10.1002/epi4.12887

**Published:** 2024-01-05

**Authors:** Denis M. Nyaga, Michael S. Hildebrand, Guillem de Valles‐Ibáñez, Ngaire F. Keenan, Zimeng Ye, Christy W. LaFlamme, Heather C. Mefford, Mark F. Bennett, Melanie Bahlo, Lynette G. Sadleir

**Affiliations:** ^1^ Department of Paediatrics and Child Health University of Otago Wellington New Zealand; ^2^ Department of Medicine (Austin Health) University of Melbourne Melbourne Victoria Australia; ^3^ Murdoch Children's Research Institute Royal Children's Hospital Melbourne Victoria Australia; ^4^ Center for Pediatric Neurological Disease Research St. Jude Children's Research Hospital Memphis Tennessee USA; ^5^ Population Health and Immunity Division The Walter and Eliza Hall Institute of Medical Research Parkville Victoria Australia; ^6^ Department of Medical Biology University of Melbourne Parkville Victoria Australia

**Keywords:** genetic testing, *PAFAH1B1*, *SCN1A*, SNP arrays, structural variants, whole‐genome sequencing

## Abstract

About 50% of individuals with developmental and epileptic encephalopathies (DEEs) are unsolved following genetic testing. Deep intronic variants, defined as >100 bp from exon–intron junctions, contribute to disease by affecting the splicing of mRNAs in clinically relevant genes. Identifying deep intronic pathogenic variants is challenging and resource intensive, and interpretation is difficult due to limited functional annotations. We aimed to identify deep intronic variants in individuals suspected to have unsolved single gene DEEs. In a research cohort of unsolved cases of DEEs, we searched for children with a DEE syndrome predominantly caused by variants in specific genes in >80% of described cases. We identified two children with Dravet syndrome and one individual with classic lissencephaly. Multiple sequencing and bioinformatics strategies were employed to interrogate intronic regions in *SCN1A* and *PAFAH1B1*. A novel de novo deep intronic 12 kb deletion in *PAFAH1B1* was identified in the individual with lissencephaly. We showed experimentally that the deletion disrupts mRNA splicing, which results in partial intron retention after exon 2 and disruption of the highly conserved LisH motif. We demonstrate that targeted interrogation of deep intronic regions using multiple genomics technologies, coupled with functional analysis, can reveal hidden causes of unsolved monogenic DEE syndromes.

**Plain Language Summary:**

Deep intronic variants can cause disease by affecting the splicing of mRNAs in clinically relevant genes. A deep intronic deletion that caused abnormal splicing of the *PAFAH1B1* gene was identified in a patient with classic lissencephaly. Our findings reinforce that targeted interrogation of deep intronic regions and functional analysis can reveal hidden causes of unsolved epilepsy syndromes.

**Key points**

- Deep intronic variants, defined as >100 bp from exon–intron junctions, contribute to disease by affecting the splicing of mRNAs in clinically relevant genes.
- Identifying deep intronic pathogenic variants is challenging, and interpretation is difficult due to limited functional annotations.
- For individuals with a DEE phenotype highly congruent with a specific gene, targeted interrogation of deep intronic regions using multiple genomics technologies and functional analysis can identify missed pathogenic variants.

## 1 INTRODUCTION

Developmental and epileptic encephalopathies (DEEs) are a group of severe epilepsies where neurodevelopmental impairment is due to not only the underlying etiology but also the negative impact of epileptic activity.^1^ These syndromes are predominantly genetic and typically caused by de novo single nucleotide variants (SNVs), insertions and deletions (indels), and large structural variants (SVs).^1^ Despite over 800 associated DEE genes,^2^ a genetic diagnosis is only made in half of the individuals.^1^ Children with a specific DEE syndrome typically have a private variant within one of the multiple genes known to cause that syndrome.^1^, ^3^ Genome‐wide genetic testing including high‐resolution analysis of copy number variants is therefore recommended for children with DEE.^3^

There are a small number of DEE syndromes, such as Dravet syndrome, which are predominantly monogenic disorders due to pathogenic variants in a specific gene in over 80% of children.^1^, ^3^ It is likely that in some of the unsolved individuals with these DEEs, pathogenic variants, particularly those within deep intronic regions, are not being identified with present sequencing and bioinformatic strategies.^4^ Even if identified, determining the pathogenicity of these deep intronic variants, defined as variants located >100 bp away from exon–intron boundaries, is challenging due to the limited functional annotations making interpretation difficult.^4^

Here, we apply comprehensive genetic analysis to three children suspected to have monogenic DEE phenotypes and identify a novel and pathogenic de novo deep intronic 12 kb variant in *PAFAH1B1* in a DEE due to classic lissencephaly.

## 2 METHODS

### 2.1 Cohort

In a New Zealand research cohort of children with DEEs, 89 remained unsolved despite genetic testing with single nucleotide polymorphism (SNP) arrays, exome (WES), and genome sequencing (WGS). The sequencing data had been previously analyzed for exonic SNVs, indels, and SVs within established epilepsy genes.^2^ The presence of a DEE and the specific epilepsy syndrome was diagnosed based on the International League Against Epilepsy (ILAE) criteria,^5^, ^6^ using parental interview, medical records review, examination, MRI, and EEG findings for each child.

Within this unsolved DEE cohort, we searched for individuals with DEE syndromes known to be caused by variants in specific genes in >80% of cases. This led to the identification of three children: two with Dravet syndrome, caused by variants in *SCN1A* in >90% of children,^3^ and one with DEE associated with classic posterior to anterior severity lissencephaly, caused by variants in *PAFAH1B1* in >80% of children.^7^ We employed a secondary analysis to intensively interrogate the deep intronic regions of these genes in these children.

The New Zealand Health and Disability Ethics Committee approved the study. Informed consent was obtained from parents.

### 2.2 Molecular genetic methods

#### 2.2.1 Standard analysis

DNA was extracted from blood. Details of the initial sequencing and analysis are described in Data S1. Briefly, samples were genotyped using the Illumina GSA‐MD v1.0. CNV calling, annotation, and filtering were performed. WES and WGS were performed. SNVs and indels were annotated and filtered. SV calling on WGS was performed using 3 methods, namely, CNVnator v0.4.1, Manta v1.6.0, and Smoove v0.2.8 (Data S1). SVs from individual pipelines were merged and SVs were retained if called by ≥2 methods. The final SV calls were annotated and filtered (Data S1).

#### 2.2.2 Secondary analysis for the identification of intronic variants

We examined all variants in *SCN1A* and *PAFAH1B1*, even if they failed our standard analysis. Intronic data from SNP arrays and WGS was further interrogated.

Deep intronic SNVs and indels obtained from WGS were annotated using the SpliceAi tool^8^ to assess potential splicing effects.

Deep intronic CNVs in SNP arrays, which were previously dismissed due to their size and SNP count being less than the suggested threshold of >20 kb and > 20 SNPs,^9^ were evaluated. PennCNV v1.05 (Data S1) generated graphical representations of these deep intronic CNVs using log R ratio (LRR) and B allele frequency (BAF) data. Deep intronic SVs identified from WGS, previously filtered out due to detection by only a single pipeline, were selected for additional scrutiny. The Samplot tool v1.3.0 (https://github.com/ryanlayer/samplot) generated plots of these intronic SVs from WGS, verifying sequencing coverage.

Plausible intronic variants were segregated, utilizing droplet digital PCR (ddPCR) for SVs (Data S1), while Sanger sequencing was employed for the examination of SNVs and indels.

#### 2.2.3 RNA sequencing analysis

We performed RNA sequencing and functional analysis on whole blood RNA from three controls and the patient with an identified intronic variant. Sequencing analysis was performed using the nf‐core/rnaseq v3.9.0 pipeline (Data S1). Quantification of the intron retention (IR) events was performed using IRFinder‐S v2.0.1 (Data S1). Differential RNA expression analysis was performed with the built‐in DESeq2 v1.28.0 software of the nf‐core/rnaseq pipeline (Data S1).

## 3 RESULTS

**FIGURE 1:**
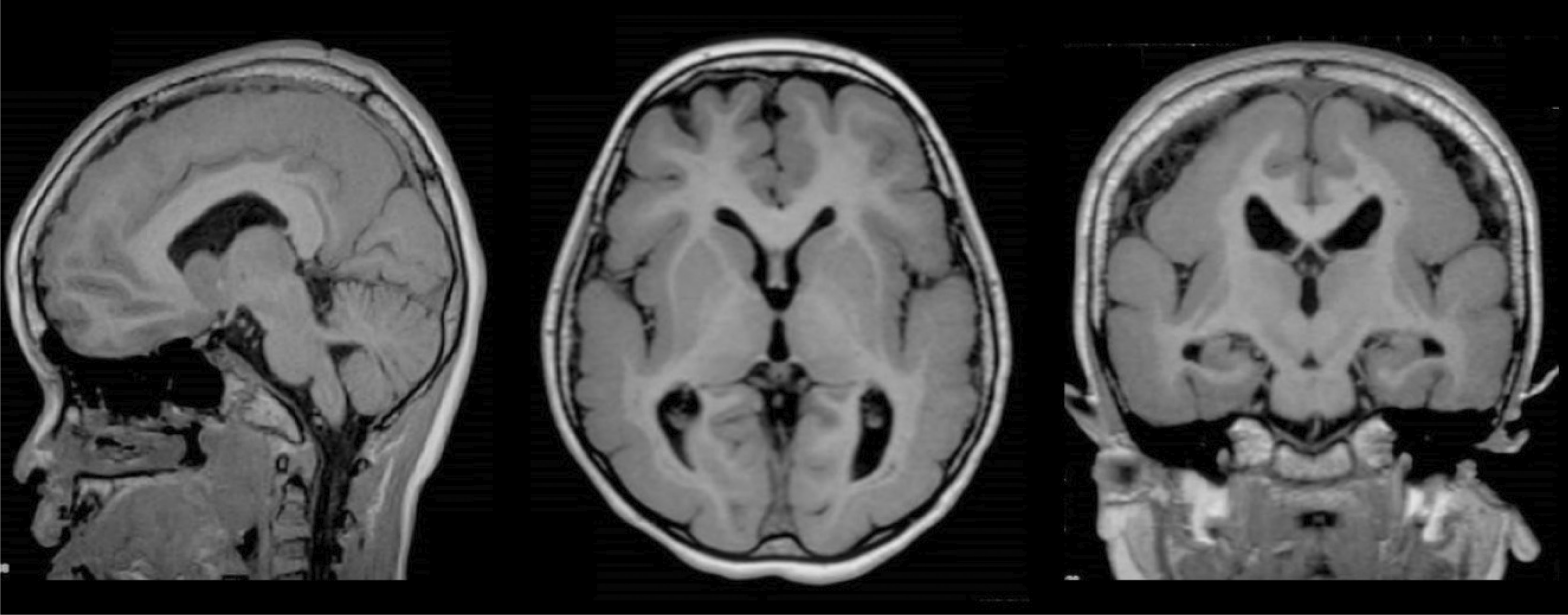
MRI at 8 years of age. Sagittal, axial, and coronal T1 images showing extensive pachygyria particularly in the parietal and occipital lobes. The corpus callosum, basal ganglia, brainstem, and cerebellum are normal.

**FIGURE 2:**
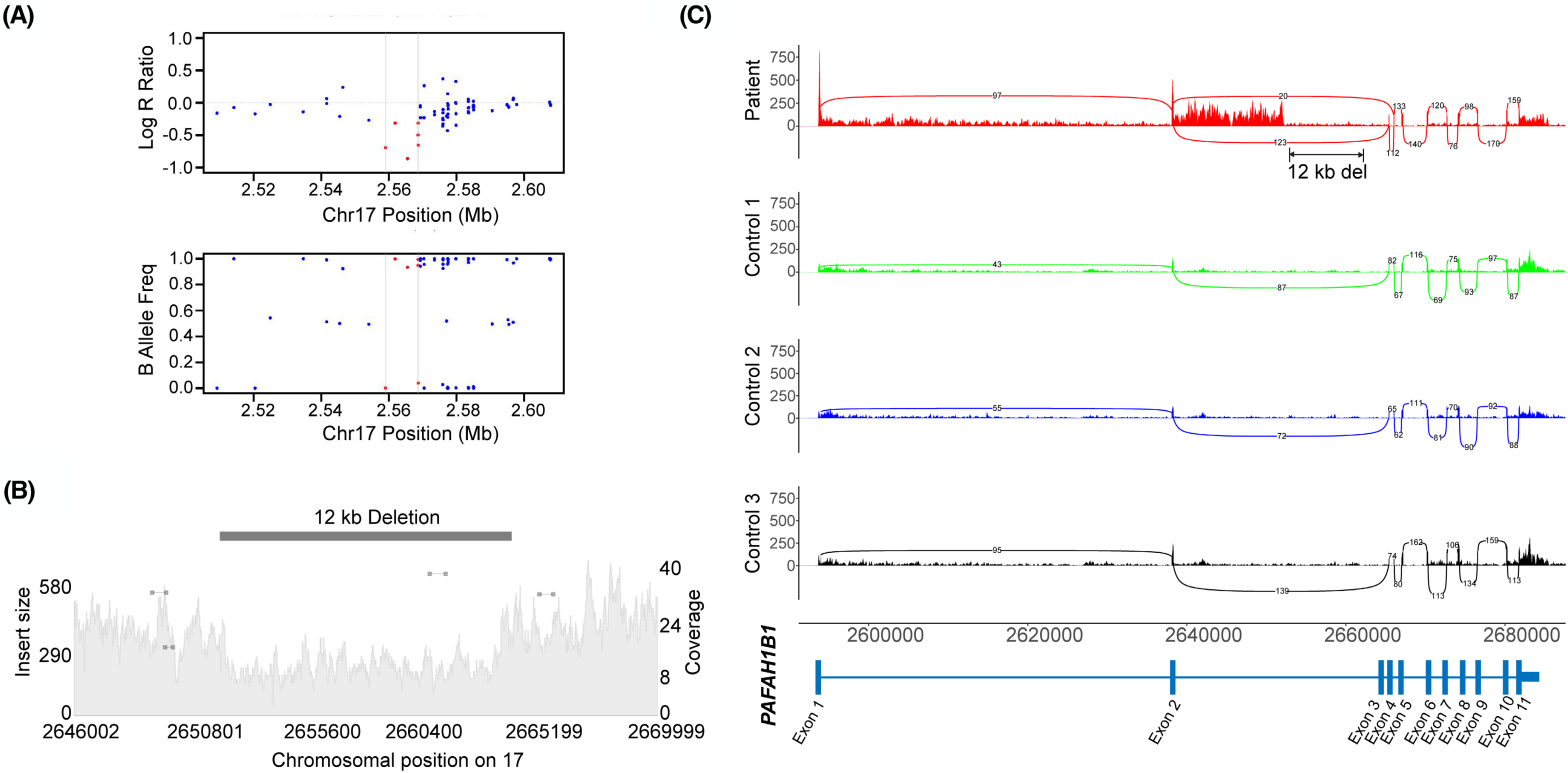
The 12 kb deep intronic deletion in *PAFAH1B1* causes aberrant splicing. (A) Log R ratio (normalized total signal intensity; LRR [top panel]) and B allele frequency (normalized allelic intensity ratio; BAF [bottom panel]), which were used to detect the deletion through PennCNV analysis of Illumina GSA v1.0 SNP arrays. The red dots indicate the markers inside the CNV call (chr17:2655725–2665395 [hg38]), but the markers were less than 20, which disqualifies it as a high‐confidence CNV call. (B) Samplot of the 12 kb deletion (chr17:2652001–2664000 [hg38]) confirmed by SV analysis of the patient's WGS data. The coverage for the region is displayed with a gray‐filled background, and the drop in the depth indicates the presence of a deletion. (C) Sashimi plot displaying partial intron retention in exon 2 in the patient (red) but not in controls (blue, green, black). The double‐headed arrow (black) shows the location of the deep intronic deletion between exon 2 and exon 3, and the bottom panel (blue) illustrates the relative positions of the *PAFAH1B1* exons.

No intronic variants in *SCN1A* were identified in two children with unsolved Dravet syndrome. In the individual with DEE due to classic lissencephaly (Figure 1), no intronic variants that passed the filtering criteria were identified. However, a low‐confidence 9.6 kb deletion (chr17:2655725–2665395 [hg38]) deep within intron 2 of *PAFAH1B1* was identified by PennCNV analysis of SNP arrays. The deletion was small (<20 kb) and supported by only six SNPs (<20 SNPs; Figure 2A). This SV was also present in the WGS as a 12 kb deletion (chr17:2652001–2664000 [hg38]) and located 13.6 kb away from the splice donor at exon 2 and 1.3 kb from acceptor at exon 3 (Figure 2B). The deletion was only identified by the CNVnator and not by the other two SV callers, Manta and Smoove. ddPCR experiments confirmed the de novo origin of the deletion.

### 3.1 RNA splicing analysis of the deep intronic microdeletion in *PAFAH1B1*

The deep intronic deletion caused partial intron retention after exon 2 (Figure 2C). IRFinder‐S indicated a “NonUniformIntronCover” and “LowSplicing” warning specifically in the second intron of the patient but not in the control samples (Table S1). In addition, the *PAFAH1B1* intron of the patient had an IR ratio of 0.29 and intron depth of 28, while the controls had an IR ratio of <0.2 and intron depth of <1 consistent with intron retention in the patient. Differential RNA expression analysis performed using DESeq2 v1.28.0 (Data S1) indicated that the patient has a distinct gene expression profile for *PAFAH1B1* compared to controls (Figure S1).

### 3.2 Clinical data of the individual with a DEE due to isolated lissencephaly

This 26‐year‐old woman had severe ID and a DEE secondary to lissencephaly. Following a normal pregnancy and birth, she was noted to have delayed development at 5 months and lost the ability to grasp items and sit independently at 8 months. She developed epileptic spasms at 8 months. Epileptic spasms, tonic and tonic–clonic seizures are drug‐resistant to 11 antiseizure medications. EEG showed multifocal slowing with frequent multifocal and generalized spike and wave enhanced by sleep. She can speak a few words and finger feed but cannot walk independently. She is hypotonic with decreased reflexes. MRI shows classic lissencephaly with extensive pachygyria, particularly affecting the parietal and occipital lobes (Figure 1).

## 4 DISCUSSION

In a cohort of children with unsolved DEEs, we found a deep intronic variant in one out of three who had a predominantly monogenic phenotype. This individual with classic lissencephaly had a deep intronic variant in *PAFAH1B1*. The variant, which is the first deep intronic variant to be reported in *PAFAH1B1*, was initially not identified using our standard bioinformatic strategy as it failed the filtering criteria for PennCNV analysis of SNP array data due to only covering 6 markers^9^ and was not identified by ≥2 WGS SV pipelines.^10^ We identified this variant using a secondary analysis, focused solely on the previously implicated genes for that disorder, to examine all suggestive variants, even if they failed the standard analysis.

Lissencephaly is a group of cortical malformations caused by abnormal neuronal migration during embryonic development in which children often present with a DEE.^7^ Although there are more than 31 lissencephaly‐related genes, up to 87% of patients with classic thick posterior to anterior gradient lissencephaly harbor causal variants in *PAFAH1B1*, the first gene identified in lissencephaly.^7^, ^11^ Individuals with *PAFAH1B1*‐related lissencephaly typically have drug‐resistant seizures (often including infantile‐onset epileptic spasms), severe intellectual disability, and early mortality.^11^

*PAFAH1B1* encodes a subunit of a brain platelet‐activating factor acetylhydrolase, which plays a critical role in neuronal migration and contributes to microtubule dynamics essential for neuronal proliferation.^11^ *PAFAH1B1* variants are typically de novo, with the majority being either single nucleotide (78%) or small genomic deletions (20–49%).^11^ About 80% of variants result in a truncated protein.^11^ Intronic SNVs, <10 bp from the intron–exon junctions, have been reported, including a recurrent variant (c.569‐10T>C) that was demonstrated to cause aberrant splicing.^12^ No deep intronic variants (>100 bp away from exon–intron boundaries) have been reported in this gene.

Our deep intronic deletion lies between exons 2 and 3, which form the highly conserved LisH motif important for regulating microtubule dynamics necessary for neuronal growth.^13^ The deletion includes an enhancer region and CTCF binding site, which are important for genome organization and gene expression. RNA sequencing showed the deletion resulted in partial intron retention and truncation of the mRNA, consequently disrupting the LisH motif. This would be expected to result in an abnormal Lis1 protein and therefore likely responsible for the patient's lissencephaly.

WGS is the sequencing of choice for detecting deep intronic variants as WES will only provide data on intronic variants close to the exon boundaries, and high‐resolution SNP arrays will not detect intronic SV of intermediate sizes, 50 bp–20 kb.^4^, ^14^ WGS was crucial in this case to confirm the causal SV.^14^ Recent studies have highlighted the importance of comprehensively investigating intronic sequences to identify deep intronic causal variants.^15^, ^16^, ^17^ Determining the splicing effects of SNV and indels within introns can be achieved using reverse transcription‐PCR (RT‐PCR) and Sanger sequencing as has been demonstrated in DEEs due to *DMN1*, *SCN1A*, and *JAM3*.^15^, ^16^, ^17^ Identifying and validating deep intronic structural variants is challenging and requires RNA/cDNA/protein‐based functional studies to understand the clinical significance of such variants.^18^

Unfortunately, due to the difficulty in determining the pathogenicity of intronic variants and the large number of DEE‐associated genes (i.e., >800),^2^ it is impractical to comprehensively interrogate and interpret intronic regions in all individuals with DEE. However, for individuals with a DEE phenotype highly congruent with a specific gene, comprehensive examination of the introns for deep variants is likely more rewarding and, therefore, should be considered. Dravet syndrome, which is due to *SCN1A* variants in >80% of individuals,^3^ is the epitome of this and not surprisingly where intronic discoveries have been made.^19^ It was the search for variants in highly conserved areas within *SCN1A* introns that led to the discovery of pathogenic splicing variants in “poison exons.”^19^ Subsequently, splicing reporter assays have been developed to functionally characterize deep intronic variants in *SCN1A*.^20^ Our deletion in *PAFAH1B1* is not in a highly conserved region, distinguishing it from variants identified in the so‐called “poison exons” in *SCN1A*.

Although our study is limited by the small number of individuals with unsolved monogenic DEE phenotypes in our cohort, our finding of a deep intronic variant in one of three children is intriguing. It illustrates the importance and effectiveness of interrogation of the deep introns in the sequencing of children with DEEs that have a strong phenotype–genotype correlation. Further studies with larger cohorts of individuals with unsolved monogenic DEE phenotypes will be able to better define the contribution of deep intronic variants to the genetic architecture of these disorders.

## AUTHOR CONTRIBUTIONS

Denis M. Nyaga: Conceptualization (equal); data curation (equal); formal analysis (lead); investigation (equal); methodology (equal); software (lead); visualization (equal); writing – original draft (lead), writing – review and editing (equal). Michael S. Hildebrand: data curation (equal); formal analysis (equal); investigation (equal); resources (equal); writing – review and editing (equal). Guillem de Valles‐Ibáñez: data curation (equal); formal analysis (lead); investigation (equal); methodology (equal); writing – review and editing (equal). Ngaire Keenan: data curation (equal); investigation (equal); visualization (equal); writing – review and editing (equal). Zimeng Ye: data curation (equal); formal analysis (equal); investigation (equal); writing – review and editing (equal). Christy W. LaFlamme: data curation (equal); formal analysis (equal); investigation (equal); writing – review and editing (equal). Heather C. Mefford: data curation (equal); formal analysis (equal); investigation (equal); resources (equal); writing – review and editing (equal). Mark Bennett: data curation (equal); investigation (equal); writing – review and editing (equal). Melanie Bahlo: Conceptualization (equal); data curation (equal); formal analysis (equal); funding acquisition (equal); investigation (equal); methodology (equal); resources (equal); writing – review and editing (equal). Lynette G. Sadleir: Conceptualization (lead); data curation (lead); formal analysis (lead); funding acquisition (lead); investigation (lead); methodology (lead); project administration (lead); resources (lead); writing – original draft (equal), writing – review and editing (lead).

## FUNDING INFORMATION

We gratefully acknowledge support from the Health Research Council of New Zealand, Cure Kids New Zealand, and the Estate of Ernest Hyam Davis and the Tedd and Mollie Carr Endowment Trust. Professor Melanie Bahlo is funded by an NHMRC Senior Investigator Grant [APP1195236]. This work was also supported by the Victorian Government's Operational Infrastructure Support Program and the NHMRC Independent Research Institute Infrastructure Support Scheme (IRIISS).

## CONFLICT OF INTEREST STATEMENT

Prof. Sadleir receives funding from the Health Research Council of New Zealand and Cure Kids New Zealand. She is a consultant for the Epilepsy Consortium and has received travel grants from Seqirus and Nutricia. She has received research grants and consultancy fees from Zynerba Pharmaceuticals and has served on Takeda and Eisai Pharmaceuticals scientific advisory panels. The remaining authors have no conflict of interest to disclose. We confirm that we have read the journal's position on issues involved in ethical publication and affirm that this report is consistent with those guidelines.

## ETHICS STATEMENT

The study was approved by the New Zealand Health and Disability Ethics Committee.

## PATIENT CONSENT STATEMENT

Participating individuals, or their parents if minors, provided written informed consent.

## Supporting information


Data S1.

Table S1.
