# Supplementary material for: Leveraging multiple approaches for the detection of pathogenic deep intronic variants in developmental and epileptic encephalopathies: A case report
Source: Epilepsia Open. 2024 Jan 5;9(2):758–64. doi: 10.1002/epi4.12887 (PMC10984288; doi:10.1002/epi4.12887)
Supplement: Supplementary file 1 — Data S1. [file EPI4-9-758-s002.docx]

**Supplemental Information**

**Molecular Genetic Methods**

*SNP array genotyping analysis*

Samples were genotyped on the GSA-MD v1.0 (Illumina, San Diego) using standard procedures on genomic DNA derived from patients’ whole blood. Signal intensity files containing SNP name, chromosome, position, genotype, Log R Ratio (LRR) and B Allele Frequency (BAF) for all markers were prepared from the VCFs (based on human genome assembly [hg19]) provided using a custom script. Copy number variants (CNVs) calling was performed using PennCNV (v1.05) CNV calling function (detect_cnv.pl) as previously described to detect CNVs only on autosomal chromosomes.^1^ PennCNV implements a hidden Markov model (HMM) for kilobase-resolution detection of CNVs by incorporating the allelic intensity ratio of each SNP marker, together with the total signal intensity, SNP allele frequency, and the distance between neighbouring SNPs.^1^ Adjacent CNVs of the same type (*i.e*., gain or loss) in the same samples were merged using the built-in PennCNV function (clean_cnv.pl) as described previously.^1^ CNVs were filtered to retain those supported by >20 SNPs and >20 kb in length as previously described.^2^ CNVs with copy numbers of 0 and 1 were annotated as copy number loss, 2 as diploid copy number, and 3, 4, 5, and 6 as copy number gain. AnnotSV v3.2 was used to annotate CNV calls by providing population-level AF, regulatory and clinically relevant information to interpret of CNV potential pathogenicity.^3^ Finally, CNVs were filtered to exclude those overlapping known benign genomic regions reported in population-level databases (*i.e*., gnomAD, 1000 genomes ClinVar, Decipher, and Deciphering Developmental Disorders [DDD]). CNVs that overlapped with established epilepsy genes^5^ were investigated further.

*Whole-exome sequencing (WES) analysis*

Exome sequencing (Illumina HiSeq X platform) was performed by the Broad Institute Genomic Service on genomic DNA derived from patients’ whole blood. Compressed Reference-oriented Alignment Map (CRAM) files aligned to the human genome assembly (hg38) and germline variants were generated using the Picard data-processing pipeline (Broad Institute Genomic Service). Single nucleotide variants (SNVs) and insertions and deletions (indels) were annotated using Ensembl Variant Effect Predictor (VEP).^4^ We filtered variants to retain those with an allele frequency (AF) ≤0.0001 (for dominant variants) and ≤0.01 (for recessive variants) in population databases (*i.e*., ExAC, gnomAD, 1000 Genomes phase 3) and cohort AF ≤0.1. We also restricted variant interpretation only to variants predicted to have a ‘HIGH’ or ‘MODERATE’ impact by VEP, as well as those within epilepsy genes.^5^ The human genomic variant search engine (https://varsome.com/) was used to infer potential pathogenicity using the American College of Medical Genetics (ACMG) standards.

*Whole-genome sequencing (WGS) analysis*

Genomic DNA of the patients was collected from whole blood, and WGS was performed on the Illumina NovaSeq PE150 platform (Novogene, Singapore). We employed Genome Analysis Toolkit (GATK) best practices workflow as described previously^6^ to align sequencing reads to the human genome assembly (hg38) using Burrows-Wheeler Aligner v0.7.17^7^, followed by duplicate marking and base quality score recalibration using GATK v4.2.4.1.^6^ Variant calling was then performed with GATK HaplotypeCaller and the resulting germline variants were annotated using Ensembl-VEP.^4^ SNVs and indels were filtered as described in WES analysis and the pathogenicity of variants determined using ACMG standards. In addition, structural variant (SV) calling was performed using 3 pipelines, namely CNVnator v0.4.1^8^, Manta v1.6.0^9^ and Smoove v0.2.8 (https://github.com/brentp/smoove). SVs from individual pipelines were merged and retained SVs only called by ≥2 pipelines. The final SV calls were then annotated using AnnotSV v3.2^3^, filtered and prioritised as detailed in SNP array CNVs.

*Droplet digital PCR*

We designed a custom copy number assay to confirm the presence and inheritance of the novel *PAFAH1B1* (LIS1) deletion, as we described previously.^10^ Briefly, following droplet generation using EvaGreen Supermix (Bio-Rad, Hercules, CA) samples were amplified on a C1000 Touch thermal cycler using the following cycling conditions: 95°C for 10 minutes for one cycle, followed by 40 cycles at 94°C for 30 seconds and 55°C for 60 seconds, one cycle at 98°C for 10 minutes and 12°C for infinite. Post-PCR products were read on the QX200 droplet reader (Bio-Rad) and analyzed using the QuantaSoft software. Primer sequences are available upon reasonable request.

*RNA sequencing analysis*

Sequencing analysis was performed using nf-core/rnaseq pipeline v3.9.0 as detailed in https://nf-co.re/rnaseq/usage. Reads were pre-processed using Trim Galore v0.6.7 (https://github.com/FelixKrueger/TrimGalore) and fastp v0.22.0^11^ to trim adapters and filtered based on quality. Genomic contaminants and ribosomal RNAs were removed using BBSplit (https://github.com/BioInfoTools/BBMap) and SortMeRNA (https://github.com/sortmerna/sortmerna), respectively. High quality reads were then aligned to the human genome assembly (hg38) using STAR v2.7.10a (https://github.com/alexdobin/STAR) and transcriptome quantification was performed using Salmon v1.5.2 (https://github.com/COMBINE-lab/salmon).The resulting BAM files were sorted and indexed using SAMtools v1.15.1 (https://github.com/samtools/samtools) and the Integrative Genomics Viewer (IGV; https://github.com/igvteam/igv) was used to visualise the alignments.

Quantification of the intron retention (IR) events was performed using IRFinder-S v2.0.1^12^ as demonstrated on https://github.com/RitchieLabIGH/IRFinder. Introns were considered to be retained if they had an IR ratio >0.2, intron depth >4, and the number of reads spanning the exon-exon junction (*i.e*., splice exact) >10 (Table S1).

*Differential RNA expression analysis*

Gene expression levels were log-transformed and variance-stabilized using the VST method in DESeq2 v1.28.0^13^ software of the nf-core/rnaseq pipeline, a transformation that makes the variance of the data more uniform over the mean. The Euclidean distances were calculated between each pair of samples. The resulting Euclidean distances were then visualised using a heatmap, with lower distances (indicating similar gene expression profiles) in blue and higher distances (indicating different gene expression profiles) in white. Finally, hierarchical clustering, represented by the dendrogram, grouped samples based on the similarity of their gene expression profiles. The calculated Euclidean distances highlight that our patient's sample exhibits a unique gene expression profile distinctly different from the controls (Figure S1).


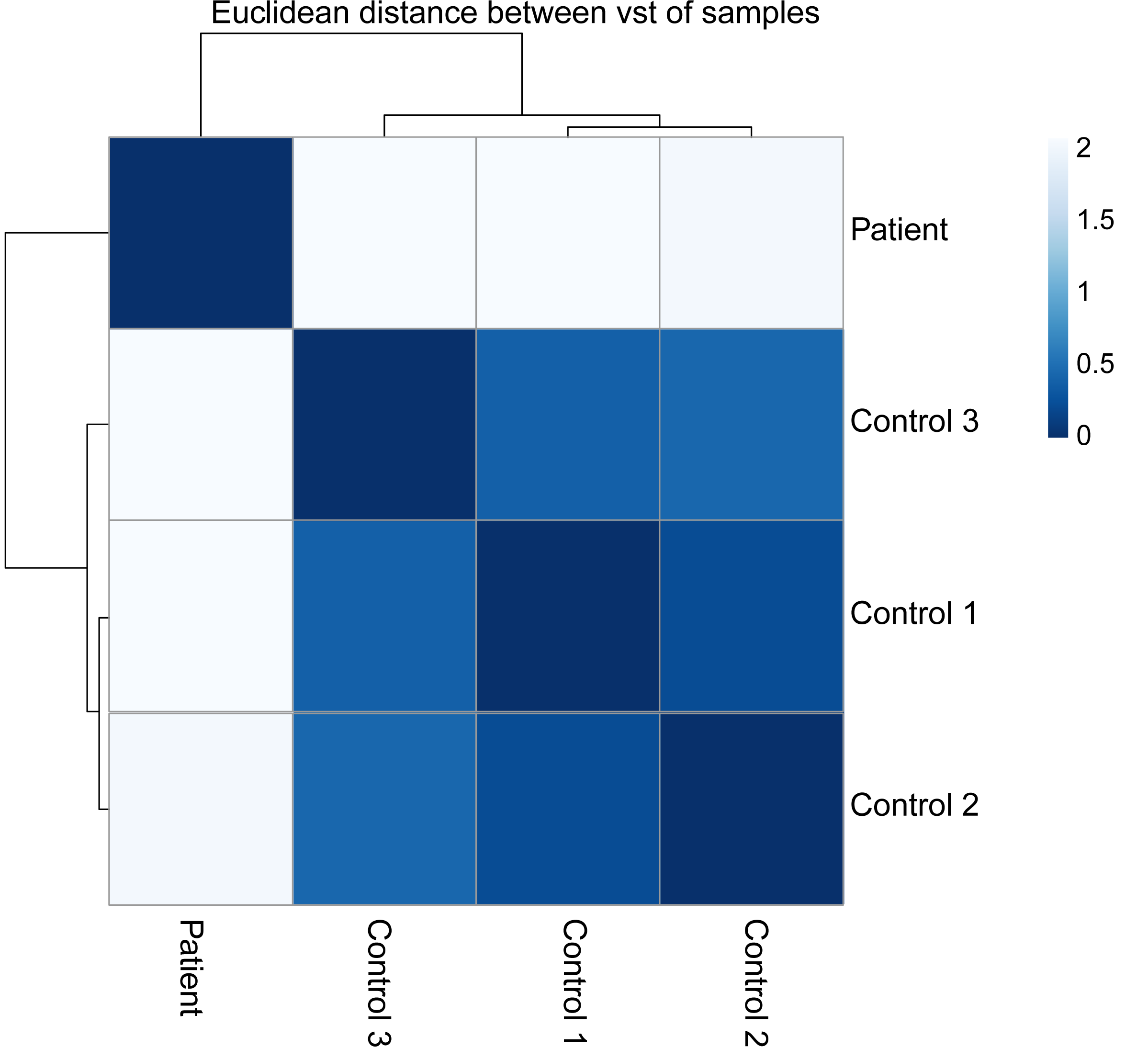


**Figure S1. The patient exhibits a distinct gene expression profile for *PAFAH1B1* compared to controls.** The figure displays the Euclidean distances between four samples in the high-dimensional space of variance-stabilized transformed (VST) *PAFAH1B1* gene expression data: three from control individuals (Control 1, Control 2, Control 3) and one from our patient with the deep intronic deletion in *PAFAH1B1* (Patient). The patient sample (white) has a distinct gene expression profile compared to the control samples (blue).

**References.**

1. Wang K, Li M, Hadley D, Liu R, Glessner J, Grant SFA, et al. PennCNV: An integrated hidden markov model designed for high-resolution copy number variation detection in whole-genome SNP genotyping data. Genome Research. 2007;17(11):1665–74.
2. Niestroj L-M, Perez-Palma E, Howrigan DP, Zhou Y, Cheng F, Saarentaus E, et al. Epilepsy subtype-specific copy number burden observed in a genome-wide study of 17458 subjects. Brain. 2020;143(7):2106–18.
3. Geoffroy V, Herenger Y, Kress A, Stoetzel C, Piton A, Dollfus H, et al. ANNOTSV: An integrated tool for structural variations annotation. Bioinformatics. 2018;34(20):3572–4.
4. McLaren W, Gil L, Hunt SE, Riat HS, Ritchie GR, Thormann A, et al. The Ensembl variant effect predictor. 2016;
5. Oliver KL, Scheffer IE, Bennett MF, Grinton BE, Bahlo M, Berkovic SF. Genes4Epilepsy: An epilepsy gene resource. Epilepsia. 2023;64(5):1368–75. doi:10.1111/epi.17547.
6. Van der Auwera GA, Carneiro MO, Hartl C, Poplin R, del Angel G, Levy‐Moonshine A, et al. From FASTQ data to high‐confidence variant calls: The Genome Analysis Toolkit Best Practices Pipeline. Current Protocols in Bioinformatics. 2013;43(1).
7. Li H, Durbin R. Fast and accurate short read alignment with Burrows–Wheeler transform. Bioinformatics. 2009;25(14):1754–60.
8. Abyzov A, Urban AE, Snyder M, Gerstein M. CNVnator: An approach to discover, genotype, and characterize typical and atypical CNVs from family and population genome sequencing. Genome Research. 2011;21(6):974–84.
9. Chen X, Schulz-Trieglaff O, Shaw R, Barnes B, Schlesinger F, Källberg M, et al. Manta: Rapid detection of structural variants and indels for germline and cancer sequencing applications. Bioinformatics. 2015;32(8):1220–2. doi:10.1093/bioinformatics/btv710
10. Hildebrand MS, Harvey AS, Malone S, Damiano JA, Do H, Ye Z, et al. Somatic GNAQ mutation in the forme fruste of Sturge-Weber syndrome. Neurology Genetics. 2018;4(3). doi:10.1212/nxg.0000000000000236
11. Chen S. Ultrafast One‐pass FASTQ data preprocessing, quality control, and deduplication using fastp. iMeta. 2023;2(2). doi:10.1002/imt2.107
12. Lorenzi C, Barriere S, Arnold K, Luco RF, Oldfield AJ, Ritchie W. IRFinder-S: A comprehensive suite to discover and explore intron retention. Genome Biology. 2021;22(1).
13. Love MI, Huber W, Anders S. Moderated estimation of fold change and dispersion for RNA-seq data with deseq2. Genome Biology. 2014;15(12). doi:10.1186/s13059-014-0550-8.
